# Parameterizable Design on Convolutional Neural Networks Using Chisel Hardware Construction Language

**DOI:** 10.3390/mi14030531

**Published:** 2023-02-24

**Authors:** Mukesh Chowdary Madineni, Mario Vega, Xiaokun Yang

**Affiliations:** University of Houston-Clear Lake, Houston, TX 77058, USA

**Keywords:** convolutional neural network (CNN), Chisel HCL, FPGA, register–transfer level, Verilog HDL

## Abstract

This paper presents a parameterizable design generator on convolutional neural networks (CNNs) using the Chisel hardware construction language (HCL). By parameterizing structural designs such as the streaming width, pooling layer type, and floating point precision, multiple register–transfer level (RTL) implementations can be created to meet various accuracy and hardware cost requirements. The evaluation is based on generated RTL designs including 16-bit, 32-bit, 64-bit, and 128-bit implementations on field-programmable gate arrays (FPGAs). The experimental results show that the 32-bit design achieves optimal hardware performance when setting the same weights for estimating the quality of the results, FPGA slice count, and power dissipation. Although the focus is on CNNs, the approach can be extended to other neural network models for efficient RTL design.

## 1. Introduction

Neural networks (NNs) have become an extensively used technique for image classification, speech processing, digit recognition, and many more purposes [1,2,3]. In the era of high-performance computing, leveraging the design complexity, power dissipation, and quality of results is one of the big challenges for the hardware implementation of complex NNs. Field-programmable gate arrays (FPGAs) are a popular choice for hardware acceleration due to their parallelism and power efficiency, but their limited extensibility between projects and design specifications is a challenge.

Additionally, most existing FPGA implementations are based on software–hardware co-design platforms, where the processors and FPGAs are on the same chip for the execution of controllers and data processing [4,5,6]. With a wide range of applications of CNNs in image classification and detection. They can be implemented on an Electronic Control Unit (ECU) for autonomous driving assistance in an automobile application [7,8,9,10,11]. For instance, the authors in [10] discuss the application of 3D image detection for an autonomous vehicle. This 3D image analysis can be handled through CNNs by applying multiple filters on the same frame for different feature extraction. The survey in [7] highlights the acceptable accuracy and detailed spatial point extraction of CNNs for a distributed automation system. In order to provide a configurable hardware core to complex NNs, a parameterizable register–transfer level (RTL) design, which can be compatible with reference EDA tool flows and work across both FPGA and ASIC implementations, is necessary.

Another research direction for implementing NNs on FPGAs is based on generating Verilog Hardware Description Language (HDL) with HDL generators, particularly for reusable intellectual properties (IPs) such as arithmetic operators and standard design modules including wrappers and interfaces [12]. These generators are often created using script languages such as Perl/tcl or high-level synthesis tools. However, a major drawback of this approach is the lack of robust libraries supporting the generators, which can cause the generator design to be more challenging than coding in Verilog. Additionally, using such generators without considering hardware-related descriptions such as timing and performance constraints can result in timing violations and performance issues in the generated HDL code.

To leverage programming productivity and high-performance hardware implementations, using Hardware Construction Languages (HCL), such as Chisel, is one of the RTL design options [13,14,15]. Chisel HCL is an open-source, embedded domain-specific language that uses object-oriented features from Scala to construct hardware at a higher abstraction level, including hardware information such as signal width and timing. Chisel can generate RTL implementations with Verilog, which is synthesizable and can be implemented on both ASICs and FPGAs. However, previous works on HCL-related designs were limited to complex mathematical algorithms [16], thus lacking the scalability feature necessary for designing parameterizable NNs.

Under this context, this paper investigates the use of Chisel HCL for designing parameterizable convolutional neural networks (CNNs), building on prior work on the HCL–HDL design flow [17]. A case study is presented, demonstrating the design of a configurable binary design library that includes fundamental arithmetic circuits such as full-adders, full-subtractors, binary multipliers, shifters, and many more. The experimental results show that the proposed design methodology achieves the same accuracy as Verilog HDL implementations, while also estimating the hardware cost in terms of the slice count, power consumption, and maximum clock frequency. Using the design library, this paper focuses on constructing and integrating a parameterizable CNN that is open to both ASIC- and FPGA-based design tools. Specifically, the main contributions of this paper are:

- This paper presents a Verilog RTL generator for CNNs that can be easily customized by parameterizing the precision of submodules and the structural designs of layers, such as the streaming width of convolution and fully connected layers, as well as the max/mean pooling layers.
- This paper conducts a design flow from a Chisel HCL description to a Verilog HDL design and, lastly, to the final hardware cost, implementation, and evaluation. The experimental results show that the 32-bit design achieves the optimal normalized performance when considering the error percentage, FPGA slice count, and energy dissipation with equal weights.
- Our proposed streaming design achieves an accuracy of 98.39% with a lower resource cost and latency compared to prior works. By employing an iterative design structure, it can further reduce the resource cost in terms of look-up tables (LUTs) and registers at the expense of more clock cycles. The case study can be extended to the design of other complex NNs as well as multi-layer CNN designs.

The remainder of this paper is organized as follows: Section 2 introduces the related works to the application of implementing CNNs on FPGAs, and Section 3 presents the background of the CNN. In Section 4, the proposed parameterizable design of the CNN is introduced. The system integration and static analysis are further discussed in Section 5. In Section 6, the FPGA design performance is evaluated in terms of the slice count and power dissipation. Finally, Section 7 concludes this paper.

## 2. Related Works

Numerous works have focused on the hardware design of CNNs, primarily addressing challenges such as reducing computational complexity and energy dissipation [18,19]. For example, real-world applications that involve complex computation require a hardware accelerator with floating-point (FP) operations. In order to reduce the hardware cost, the accuracy can be traded off by employing fixed-point implementations for calculation [18]. As an example in [19], an 8-bit and 16-bit fixed-point design is carried out to demonstrate a lower accuracy implementation with less hardware resource cost compared with the FP implementations. Another immediate option for low-cost design is to simplify the design structures of the CNN models. For example, in [20], the authors implemented a Super Skinny CNN (SS-CNN) with 39,541 parameters and three layers in addition to the input and output layer. The overall latency of the design on a Cyclone IVE FPGA is about 2.2 s. Additionally, hardware design engines have been widely employed as accelerators for complex computations of neural networks. For example, a latency of 3.58 ms and 3.2 ms with an accuracy of 98.64% and 96% were achieved in two high-level synthesis designs on LeNet-5 CNN, presented in [21,22], respectively.

To balance the quality of the CNN results with hardware resource utilization, the RTL designs are based on the FP operators or intellectual properties (IPs) provided by the FPGA tools. For example, the FP adders and multipliers from AMD Vivado are used to construct multi-layer perceptron (MLP) NNs in [23,24]. By offering different levels of parallelism in the design engine, five MLP NNs were presented as case studies. The implementations are based on the provided Vivado IPs and not the RTL programming; therefore, the implementations are not synthesizable to be an ASIC. Second, all five design structures are based on single-precision modules due to the limited configurations provided by AMD Vivado. Another example of a configurable design was demonstrated in [25]. The RTL design on a CNN has reconfigurable convolution, pooling, and fully connected modules. The system was built using reconfigurable IP cores and deployed on the Intel Cyclone10 FPGA platform. The experimental results showed that the implementation achieved a latency of 17.6 us with an accuracy of 97.57%.

From the hardware designer’s perspective, the fundamental design neurons in each layer can be constructed with multiple FP operators such as adders and multipliers. Furthermore, the CNN can be integrated with multiple neurons and network layers. In the preliminary results in [17], the binary design library was proposed as a case study of the design flow from Chisel HCL to Verilog HDL to the final FPGA development and evaluation. In this paper, the extended work focuses on the parameterizable design of CNNs with the binary and FP design library. The proposed approach supports a series of pipelined CNN engine designs that can be applied to a wide variety of problems. Highly parameterized digital circuit generators allow developers to re-use and customize their implementations for different design specifications, thus cutting design cycles and complexity.

## 3. Fundamental Theorem of Convolutional Neural Networks

This section discusses the fundamental theorem of CNNs, including the number of hidden layers and activation functions used for constructing the network.

### 3.1. CNN Structure

**Figure 1:**
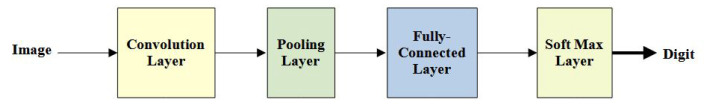
CNN Structure.

The mathematical model for a basic CNN typically includes a multi-layer convolution layer, a pooling layer, a fully connected layer, and a softmax layer, as illustrated in Figure 1. The output of each layer is referred to as the output feature map, which serves as the input feature map for the subsequent layer, creating inter-operational characteristics. The number of convolutional and pooling layers can be adjusted and arranged in the network depending on the implementation algorithms and application requirements.

### 3.2. Convolutional Layer

The convolutional layer performs feature extraction from the input matrices. The calculation of the convolution result involves the idea of both a local receptive field and shared weights and bias.

#### 3.2.1. Local Receptive Fields

**Figure 2:**
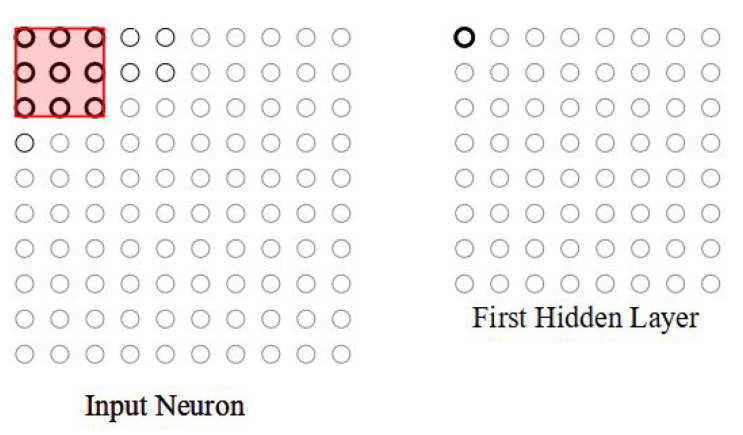
Local receptive field in the top-left corner to connect to first hidden neuron.

The convolution layer input is considered a 10×10 pixel intensity, which is fed in as input matrices. Instead of entering all the input pixels, the connections are established in small, partial regions of the input matrix. For example, a 3×3 region, corresponding to 9-input pixels, is connected to a small region of the input neurons. So, for a particular neuron, the connections are shown in Figure 2. On the right-hand side of the image, the highlighted element is the output neuron corresponding to the selected partial region on the left-hand side of the image.

**Figure 3:**
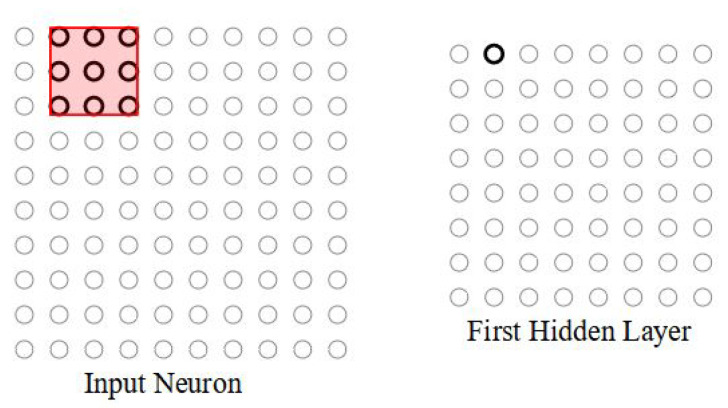
Local receptive field is slid over by one pixel to the right (i.e., by one neuron), to connect to a second hidden neuron.

The region in the input pixel image to which the neuron is connected is called the local receptive field. The neuron learns an overall bias of the field. The local receptive field is slid across the entire input image, corresponding to having a different neuron in the hidden layer. Figure 3 shows that the local receptive field is moved along the input image by one pixel at a time. The stride length decides the shift amount of the local receptive field in the input image pixel matrix.

If the input is 10×10 and the local receptive field is 3×3, then 8×8 neurons will be generated in the hidden layer. This is because the local receptive field can be moved seven neurons across (or downward) before reaching the edge of the input image. More generally, for an input of size A×A and a local receptive field of size B×B, the output matrix size can be calculated using an equation:

(1) $$output=rounddown\left(\frac{A-B}{stride}\right)+1$$

where the round down function is used to round the result to the nearest lowest integer. For example, the above input matrix A is 10×10, B is 3×3, and stride length is one. So, the output matrix size will be 8 × 8.

#### 3.2.2. Shared Weights and Biases

The shared weights and biases determine the value of the output neuron in the convolution layer. The exact weights and biases will be used by the entire matrices to obtain 8×8 hidden neurons. In other words, the output for the *j*th, *k*th hidden neuron can be expressed as:

(2) $$output_{j,k}=\sigma \left(b_{j,k}+\sum_{l=0}^{2}\sum_{m=0}^{2}w_{l,m}a_{j+l,k+m}\right).$$

where σ represents the neural activation function, such as the Rectified Linear Unit (ReLU) activation function [25]. The indexes *j* and *k* range from 0 to 2 for a 3×3 local receptive field. $b_{j,k}$ represents the shared value for the bias, $w_{l,m}$ denotes a 3×3 array of shared weights, and $a_{x,y}$ denotes the input activation at position x, y. The equation indicates that all the neurons in the hidden layer detect the same feature. It is generally called the feature map from the input layer to the hidden layer.

### 3.3. Pooling Layer

The pooling operation can be carried out after the convolution layer, which prepares a condensed feature map. Each unit in the pooling layer may summarize a region of 2×2 neurons from its previous layer. The operation used in summarizing the output is max-pooling or mean-pooling.

**Figure 4:**
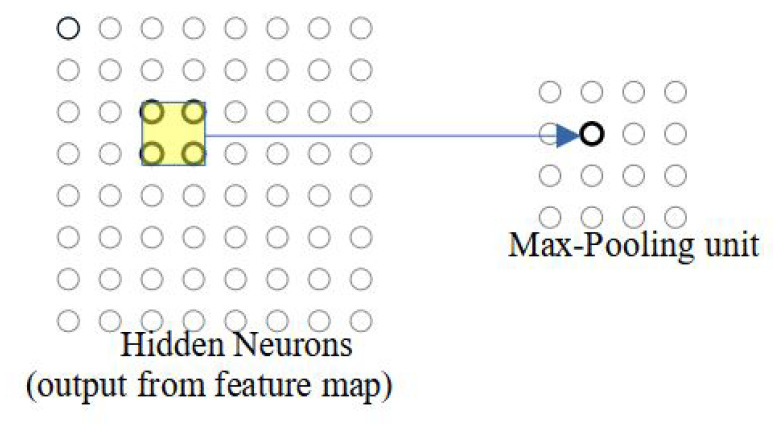
Max pooling of 2 × 2 on the output feature map.

In the max-pooling, the pooling region outputs the maximum activation in the 2×2 input region, as illustrated in Figure 4. On the left-hand side, it shows the output neurons of the previous layer. The maximum value in the selected region is processed as an output of the max-pooling operation. In contrast, in mean-pooling, the region outputs the average of the four neurons in the selected region. The selection of the max or mean pool operation is parameterizable in our proposed work. Practically, the max pool operation extracts the brightest pixel in the regions, whereas the mean pool operation spreads the brightness of pixels in the given area.

As shown in this example, the input to the pooling layer is 8×8 neurons and the stride is two; therefore, using Equation (1), the size of the output matrix is 4×4.

### 3.4. Fully Connected Layer

**Figure 5:**
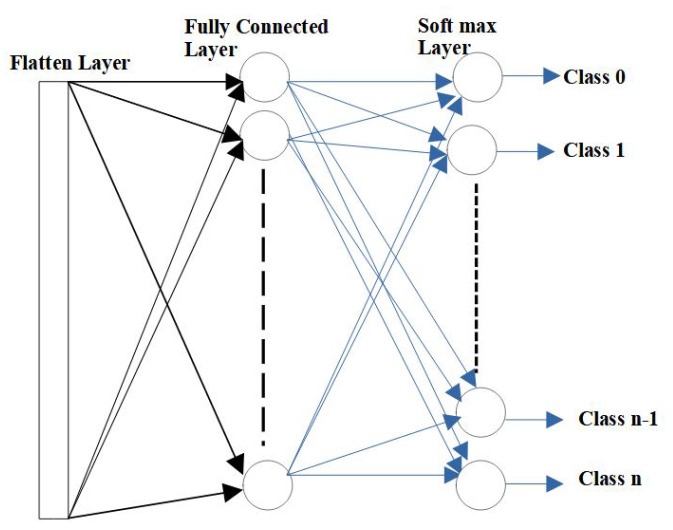
Fully connected layer operation.

After the convolutional and pooling layers, the input matrix is converted into a suitable form for CNN. In what follows, the matrix is further flattened into a column vector. The linear output is fed into a feed-forward neural network. In fully connected layers, every input is connected to every output by a learnable weight. Figure 5 shows the operation of a fully connected layer after flattening the output from the pooling layer. In the fully connected layer, each neuron performs a multiplication–addition operation with the kernel weights and bias values, which is similar to the execution in the convolution operation.

### 3.5. Softmax Layer

In the softmax layer, the output corresponds to the highest value in the output neurons from the fully connected layer. In the application of digit recognition, as an example, this layer is used to find the highest percentage of the classified results. This method of distinguishing between the dominating and low-level features in an image is called the softmax classification technique.

## 4. Design on Parameterizable CNNs

This section discusses the idea of designing a parameterizable CNN using Chisel HCL. The IntelliJ software from JetBrains is employed as the developing tool using Scala plugins and Chisel HCL libraries. By parameterizing the proposed generator, the RTL design on the CNNs can be constructed. The design functionality is verified using a direct test from Chisel HCL, and the corner test cases are tested through RTL verification using Siemens ModelSim. After verification, the experimental results, including the slice count, latency, and power cost, are estimated using AMD Vivado.

### 4.1. Convolution Module Design

The convolution layer is the most computationally complex part of the CNN, which is used to extract the features of the input matrices using kernel filters or convolution kernels. The specific goal is to convolve the input matrices with a kernel filter, weigh the summation of the convolution results, and then obtain the output feature map of this layer after processing it through an activation function.

To implement Equation (2) with code, four layers of embedded “for loop” are needed. Here, pseudo-codes as Algorithm 1 are adopted to output the 64 neurons from the convolution layer. An element-wise product between each element of the kernel and the input matrix is calculated at each location of the matrix and summed to obtain the final value in the corresponding position of the feature map. The convolution operation involves two-dimensional multiplication–addition calculations. An element-wise product between each element of the kernel filter and the input matrix is calculated at each location of the matrix and summed to obtain the final value in the corresponding position of the output feature map.
**Algorithm 1:** Convolution layer computation   Input: input_A: Input data of image pixel input_B: Kernel map array   Output: conv_out: Output feature map array Algorithm:   for(i=0;i<in_matA_size-2;i++)//Periodically traverse the rows of input   for(j=0;j<in_matA_size-2;j++)//Periodically traverse the columns of input   for (n=0;n<in_matB_size;n++)//Periodically traverse the rows of kernel map   for(m=0;m<in_matB_size;m++)//Periodically traverse the columns of kernel   map   output = input_A[i× stride+j] × input_B[n+m]   conv_out=(output<0)?0:output;

#### 4.1.1. Streaming Design on Convolution Module

For the hardware implementation of the convolution layer, various methods have been proposed. As an example, [25] presented a conventional method by implementing convolution hardware with a 3×3 kernel circuit. The implementation fully utilized the parallelism for computing the convolution results. The convolution window sliding was realized, and a convolution computation circuit of the efficient parallel pipeline operation was formed. The downside of such implementations is that the design structures have to employ a fixed length of convolution.

**Figure 6:**
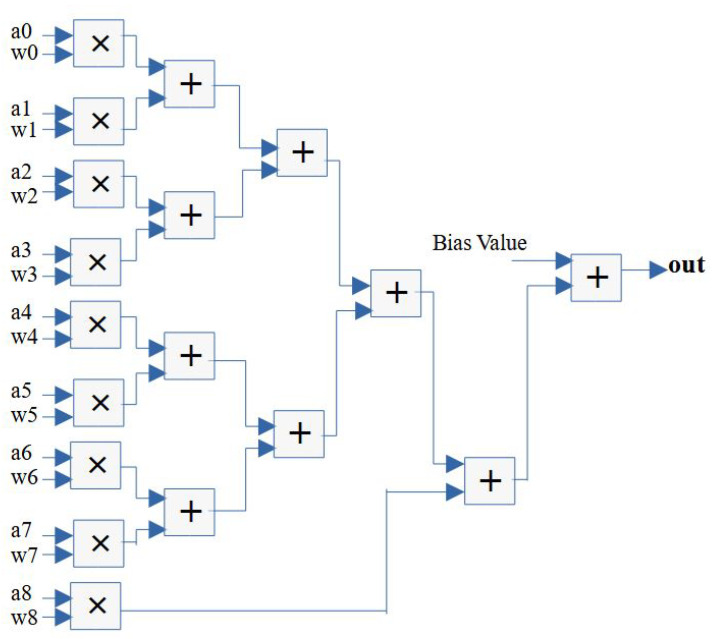
Streaming design structure of a convolutional layer neuron.

In this paper, therefore, a pipelined structure and reconfigurable convolution module is presented. By parameterizing the streaming width of the design, different convolution modules with Verilog HDL can be generated. As a case study, Figure 6 shows the design module that adopts a stream of nine inputs (3×3) for every clock cycle and calculates the convolution result within seven clock cycles, during which the following stream of inputs is adopted. Specifically, the nine input data (denoted as “a0-a8”) and weights (denoted as “w0-w8”) are fed into the design engine in parallel, and, then, each FP operator including the FP multiplier and FP adder adopts one clock cycle for the computation. As a result, the design structure utilizes nine FP multipliers and nine FP adders.

**Figure 7:**
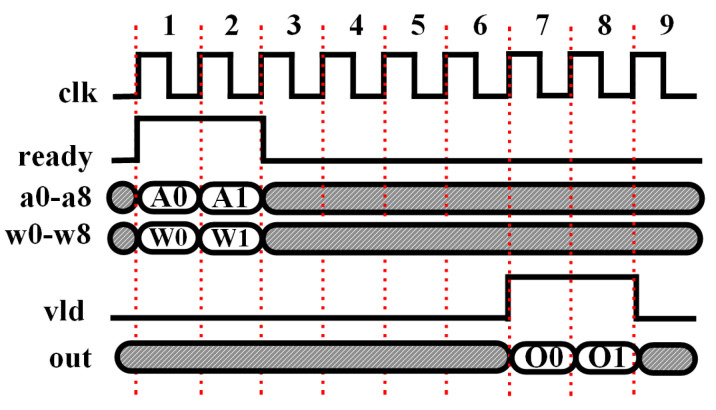
Timing diagram of streaming design of a convolutional layer neuron.

As the timing diagram shown in Figure 7, in the first and second clock cycles, two consecutive groups of inputs (denoted as “A0” and “A1”) are fed into the engine, which is specified by an asserted “ready” signal to indicate the valid data input. Notice that “A0” and “A1” are two groups of inputs, and each of them includes all the nine inputs from “a0” to “a8” in parallel. Their corresponding weights (denoted as “W0” and “W1”) are read out from a ROM and the multiplications (A0×W0 or A1×W1) are performed in the same clock cycles. In the following clock cycles from two to six, the products will be pushed out and then, cascading, summed up together. Adding the bias requires one more clock cycle to push out the final groups of output (denoted as “O0” and “O1”), which are indicated by the asserted “vld” signal in the seventh and eighth clock cycles.

#### 4.1.2. Parameterizable Streaming Width on Convolution Module

As mentioned earlier, the proposed work allows for the parameterization of the streaming width *N*, enabling the generation of Verilog designs with different resource costs and latencies. Generally, the latency of the streaming design structure can be calculated as follows:

(3) $$Latency\_Streaming=1+1+roundup(\log_{2}N)+1$$

where one cycle is needed for the valid data fed into the engine, followed by an additional one cycle for the multiplications in parallel, followed by the logarithm result for the clock cycles needed by the cascading additions, and, finally, one clock cycle for the addition with the bias. The roundup(x) functions round the *x* up to the integer if $\log_{2}N$ is a fraction. For example, N=9 so that $roundup(\log_{2}9)=4$ and the final estimated result is seven clock cycles. Similarly, the number of FP operators can be approximately estimated as *N* FP multipliers and *N* FP adders for the streaming design.

**Figure 8:**
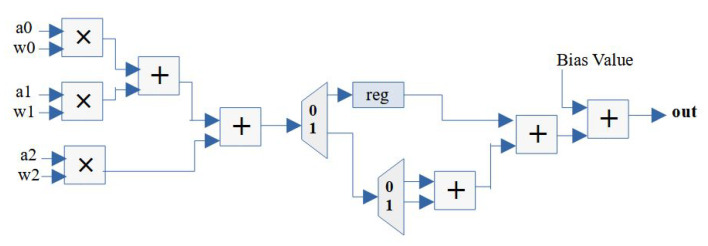
Iterative design structure of a convolutional layer neuron.

In order to reduce the number of FP operator utilization, the iterative design can be extended with less streaming width compared to the number of input data. For example, the number of data inputs is nine and the streaming width of the design is three. Thus, one group of data should be divided into three data frames and then fed into the engine over three clock cycles. The design structure is shown in Figure 8, including a register to delay one more clock cycle on the first data frame and two FP adders for accumulating the three summations from the three data frames. Finally, an FP adder is needed to add the bias to push out the final result. Notice that, for the multiplication–addition design, it only requires three FP multipliers and two FP adders that can be reused by three data frames over multiple clock cycles. The latency of the iterative design can be calculated as

(4) $$Latency\_Iterative=SW+roundup(\log_{2}SW)+SW+1$$

where SW represents the parameter of the streaming width. The first SW indicates the clock cycles for feeding in the entire data group, and the second SW shows the latency for accumulating the results from all the data frames; a final clock cycle is needed for the bias addition, and the $roundup(\log_{2}SW)$ function is used for calculating the clock cycles for the cascading summation.

**Figure 9:**
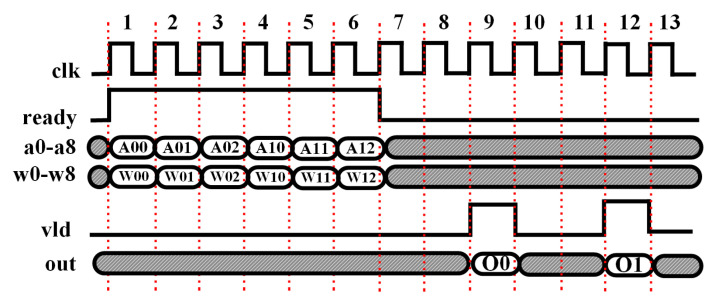
Timing diagram of iterative design of a convolutional layer neuron.

A specific example is shown in Figure 9 including two groups of data. The first group is divided into three data frames denoted as “A00, A01, and A02” and the second group is composed of three data frames denoted as “A10, A11, and A12”. Likewise, the corresponding weights are denoted as “W00, W01, and W02” for the first group data, and “W10, W11, and W12” are for the second group data. For this example, SW=3 so that the latency can be calculated as $3+roundup(\log_{2}3)+3+1=9$ clock cycles to push out the final output, where three cycles are needed for the valid data fed into the engine, two cycles for each multiplication–addition result, an additional three cycles for the accumulation of the three multiplication–addition results, and the final clock cycle for the bias value addition. Notice that the output can be pushed out over every three clock cycles in the pipeline since fewer resources are utilized for the convolution module.

### 4.2. Pooling Module

A pooling module provides a down-sampling operation that reduces the in-plane dimensions of the feature map. Unlike the convolutional layer, the pooling layers do not have any learnable parameters; instead, the filter size, stride, and padding are hyperparameters in the pooling operations. The construction of the pooling layer can be parameterized using either max pool or mean pool modules, which are implemented in this subsection.

#### 4.2.1. Max Pool Module

The most popular form of the pooling operation is the max pool, which fetches patches from the feature maps, outputs the maximum value in the filter size in each patch, and discards the rest of the values in the patch. The general filter size used in a CNN is 2×2 with a stride of two.

**Figure 10:**
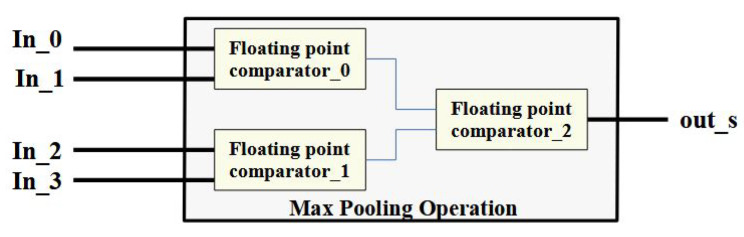
Visual representation of the max pooling using FP comparators.

Figure 10 shows the structure of the max pooling computation for a 2×2 pooling kernel. Using the FP operators, the final maximum value can be obtained by comparing all four elements. In the design of the pooling operation, the pseudo-codes adopted are shown as Algorithm 2. Implementing the max pooling operation requires four layers of embedded “for loops”. In this example, pseudo-codes are adopted to output 16 neurons from the pooling layer.
**Algorithm 2:** Max pooling computation   Input: input_A: pool_in_buff[size_A][size_B]//Input feature map array   Output: max_out: max_out_buff[size_out]//Output feature map array Algorithm:   for(i=0;i<in_matA_size/2;i++)//Periodically traverse the rows of input data   for(j=0;j<in_matA_size/2;j++)//Periodically traverse the columns of input data   for (n=0;n<2;n++)//Periodically traverse the rows of kernel map   for(m=0;m<2;m++)//Periodically traverse the columns of kernel map   tmp1=pool_in_buff[i*stride + n];   tmp2=pool_in_buff[j*stride + m];   tmp3=pool_in_buff[i*stride + 1 + n];   tmp4=pool_in_buff[j*stride + 1 + m];   max1=(tmp1>tmp2)?tmp1:tmp2;   max2=(tmp3>tmp4)?tmp3:tmp4;   max=(max1>max2)?max1:max2;   index = i*stride + j*stride + m + 8 * (in_matA_size/2 + 8);   max_out_buff[index]=max.

#### 4.2.2. Mean Pool Module

**Figure 11:**
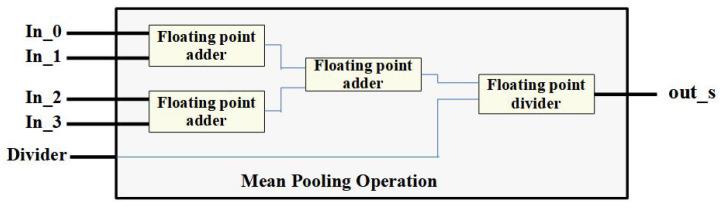
Visual representation of the mean pooling using FP adders and divider.

The mean pool technique is similar to the max pooling, where data elements are fetched from feature maps, and the output is the mean of all the elements in the fetched patch. The general filter size used in mean pooling is 2×2 with a stride of two. Figure 11 shows the implementation of the mean pooling operation using three FP adders and one FP divider for a 2×2 patch.

In the design of the pooling operation, the pseudo-code adopted is shown as Algorithm 3. Implementing the mean pooling operation requires four layers of embedded “for loops”. Similar to the algorithm shown in the design of the max pool module, pseudo-codes are adopted to output 16 neurons from the pooling layer.

From the computational design perspective, the key difference between the max pool layer and the mean pool layer is that the mean pooling operation requires an additional FP divider in the calculation of the patch mean. The downside of using mean pooling is that the pixel intensity is averaged across the region, whereas, in max pooling, only the maximum intensity value is retained. Both max pool and mean pool layers can be parameterized and generated by our proposed work, providing flexibility for constructing complex neural networks.
**Algorithm 3:** Mean pooling computation   Input: input_A: pool_in_buff[size_A][size_B]//Input feature map array   Output: mean_out: mean_out_buff[size_out]//Output feature map array Algorithm:   for(i=0;i<in_matA_size/2;i++)//Periodically traverse the rows of input data   for(j=0;j<in_matA_size/2;j++)//Periodically traverse the columns of input data   for (n=0;n<2;n++)//Periodically traverse the rows of kernel map   for(m=0;m<2;m++)//Periodically traverse the columns of kernel map   tmp1=pool_in_buff[i*stride + n];   tmp2=pool_in_buff[j*stride + m];   tmp3=pool_in_buff[i*stride + 1 + n];   tmp4=pool_in_buff[j*stride + 1 + m];   avg=(tmp1+tmp2+tmp3+tmp4)/4;   index = i*stride + j*stride + m + 8 * (in_matA_size + 8);   mean_out_buff[index]=avg.

### 4.3. Fully Connected Module and Softmax Module

After the final pooling operation, the outputs from the feature maps are flattened into a one-dimensional array of numbers and connected to one or more fully connected layers, each of which has learnable weights that enable multiplication–addition operations between every input and output. The clock cycle operations for these layers are similar to those shown in Figure 6. The implementation algorithm for the fully connected layer is similar that shown in Algorithm 1 but with different weight and bias values.

**Figure 12:**
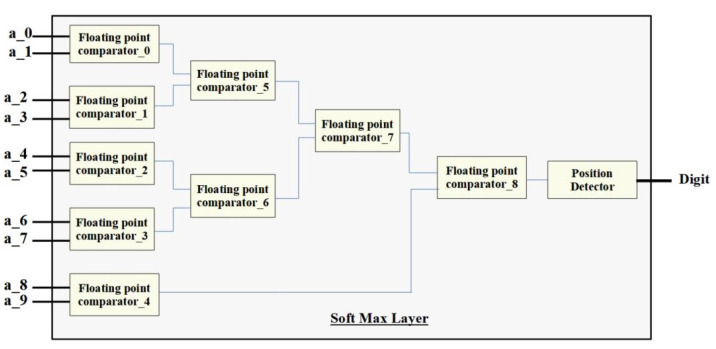
Visual representation of the softmax layer using FP comparators.

The softmax module uses FP comparators to determine the maximum value among the ten output neurons. The implementatoin algorithm for the softmax layer is shown in Algorithm 4. The design circuit shown in Figure 12 is then used to identify the position of the neuron with the highest value.
**Algorithm 4:** Softmax layer computation   Input: input_A: soft_in_buff[10]//Input feature map array   Output: Digit: Digit_buff//Output feature map array Algorithm:   for(i=0;i<in_matA_size/2;i++)//Periodically traverse the rows of input data   if(soft_in_buff[i] >= max)   Digit = i   max = soft_in_buff[i]   endif  endfor


## 5. System Construction and Static Analysis

In this section, the system-level integration and construction are further discussed. This project demonstrates the validation of the proposed parameterizable designs on CNNs. The methodology can be extended into different complex neural networks by constructing neurons with parameterizable submodules such as FP adders and multipliers, and further integrating the neurons into multi-layer networks.

### 5.1. System Design Construction

In order to construct different networks with different precision, a configurable design on the CNN is demonstrated in this section. Specifically, the network architecture can be parameterized and generated to provide different design precision including half-word, word, double-word, and quad-word. Additionally, this paper presents a parameterizable pooling design for choosing max pool or min pool operation in the generated CNN architecture. Finally, the streaming structure and iterative design of the convolution and fully connected layers are further presented. Based on the size of the local receptive field, the input parameter to the convolution is decided. Similarly, the parameters for the size of the register map are calculated upon the local receptive field and the number of computational outputs.

**Figure 13:**
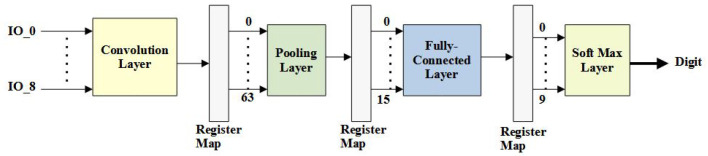
System construction on CNNs.

We follow a layered-based design style, generating Verilog HDL code for individual layers based on the number of hidden layers required for a particular application. As a case study, we employ the FP design library to build the CNN [17]. After parameterizing the precision of each FP operator and finding the design structure of each layer, the network can be constructed into a sequential system including register maps between different layers of data streams or feature maps. Figure 13 shows the register maps generated for various data widths during the generation of RTL designs.

Specifically, the proposed network uses 10×10 as the input to the convolution layer, which generates 64 neurons as the first result of the convolution operation using a 3×3 kernel filter. The convolution layer uses the ReLU activation function, defined as y=max(x,0), and the window slide step is one. Using our proposed work, the streaming width of the convolutional layer is parameterizable so that the convolution operation may require multiple clock cycles to calculate the results of the output neuron. As an example shown in this figure, the streaming design of nine inputs is configured to the implementation on the convolution layer and the results are stored in the register map before being fed into the subsequent pooling layer.

In what follows, the 64 neurons are further downsized through the pooling operation, resulting in 16 neurons using a 2×2 region map for performing the max pooling operation. The pooling layer is configurable depending on the design specifications and, by default, uses max pooling with a window slide step of two. The 16 output neurons from the pooling layer are connected to the inputs of the fully connected layer. A minimum of 10 output neurons are required for the final data classification. Hence, we use 16×10=160 weights and ten bias values for calculating a fully connected layer. The final classification result is the corresponding number of neurons with the highest value output.

### 5.2. Static Analysis of Hardware Cost

This subsection analyzes the hardware cost of different parameterizable designs presented in this paper, as shown in Table 1. In the second row, the convolutional layer design is parameterized with a streaming width of nine, and the max pooling module is selected when generating the Verilog code. The implementation with a streaming width of nine requires 45 FP multipliers (FP_MUL), 45 FP adders (FP_ADD), and 57 FP comparators (FP_COMP), and the simulation requires 100 clock cycles to classify the 10×10 inputs. Using the streaming width of three, the design in the third row shows a reduction in the utilization of FP modules but requires 2× clock cycles for data classification.

Compared with the max pool operation, the mean pool operation only utilizes nine FP comparators but much more FP adders. Similar to the difference between streaming widths of nine and three for the max pool operation, the mean pool module with a streaming width of nine requires much more resource cost in terms of FP multipliers and FP adders but spends fewer clock cycles compared with that of a streaming width of three.

Between the max and mean pool design, the mean pool operation requires FP dividers (FP_DIV) to calculate the average of the selected region, as well as more FP adders to sum the inputs. Since the resource cost of the FP adders and dividers is much higher than that of the FP compactors, the mean pool design will spend more LUTs and registers than the design of the max pool structure. Therefore, the default configuration is the max pool for our proposed work. The latency difference of the additional three clock cycles between the max and mean pool operation-based design is due to the additional three FP adder modules in the mean pool-based design.

## 6. Experimental Results

The static analysis in the previous section shows the difference between the design structures. In this section, the practical hardware performance is further estimated using AMD Vivado in terms of the quality of results, slice count, and energy cost. Vivado 2019.2 is applied as the synthesis tool with the FPGA target device Zynq UltraScale+ MPSoCs xczu19eg-ffvb1517-3-e.

### 6.1. Experiment Design

The experimental setup is based on random FP input numbers fed as an input to the convolutional layer. These random inputs are generated using Scala’s built-in random functions during the generation of the RTL code. The hardware results after the layer are displayed on the IntelliJ IDE. In our case study, we have considered our test matrix size to be 10×10, which generated a 100 random pixel intensity, and a 3×3 local receptive field that is hard coded onto the circuit. The outputs of each layer are captured and compared to the golden results generated by the software-implemented CNN using Scala language. For accuracy, multiple test results were captured for different inputs and compared with the software results.

### 6.2. Resource Cost on FPGA

After synthesis with Vivado, the hardware utilization for different designs with different precision is shown in Table 2. It can be observed that the higher resource utilization comes from the network with a higher bit width. Specifically, the 128-bit design uses more than four million LUTs and about 27 thousand registers. However, the 16-bit design only uses 9808 LUTs and 3250 registers, which is much less than the higher precision implementations. The design with less precision usually obtains less accuracy when classifying images or videos. As shown in the last column, the 128-bit design can achieve the highest accuracy and the 16-bit design obtains an accuracy with about a two percent reduction.

It concludes that a large number of resources can be saved by using low-precision designs with a slight reduction in the quality of the results. Notice that the comparison in this case study is to feed in the random matrix and monitor the output between different designs. For estimating the accuracy in real applications such as digit recognition, more error percentages would be involved by using low-precision implementations.

The number of clock cycles required for computation remains constant in spite of the increase in precision due to parallelism. The generated RTL uses a significant number of hardware resources to achieve high parallelism. The circuit consists of multiple instantiations of modules that also increase the energy consumption as discussed in the next section.

### 6.3. Energy Consumption on FPGA

In what follows, the energy dissipation is summarized in Table 3. As a result, the total on-chip energy for the 16-bit design is 0.30 mJ, including 0.28 mJ dynamic energy and 0.02 mJ static energy. As with the analysis of resource utilization, the energy dissipation for higher precision designs is significantly higher than the implementations with lower precision. The highest energy cost is 7.867 mJ, which is obtained by the 128-bit design.

### 6.4. Hardware Cost Analysis

Our proposed work can be used to evaluate the FPGA cost in the combined error percent–area–energy with a simple equation

(5) cost=(P×x)+(S×y)+E×(1−x−y).

where *P*, *S*, and *E* represent the normalized values of the error percentage, slice count, and energy consumption, respectively. Additionally, *x*, *y*, and (1−x−y) represent the weights of the three design specifications. Specifically, *x* and *y* are between 0 and 1.0; thus, the summation of all three weights would be 1.0. By configuring the three design specifications *x*, *y*, and 1−x−y, the parameterizable design can regulate the design features and target one of the design performances. For example, setting *x* and *y* as 1/3 will lead to equal weighting, and y=0 would target a design constraint with a low slice count.

**Figure 14:**
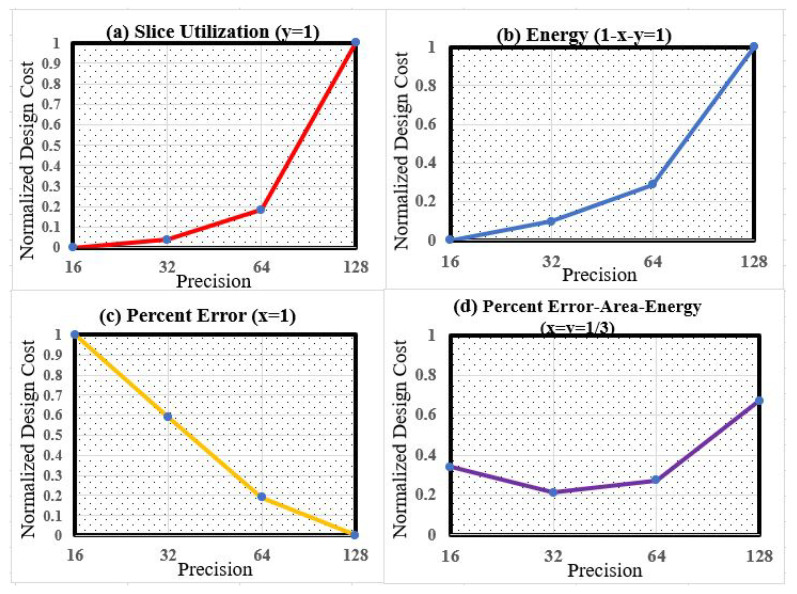
Normalized accuracy–area–energy consumption.

In Figure 14, the normalized error–area–energy design cost is summarized. When setting y=1.0, as depicted in Figure 14a, it can be observed that the 16-bit design uses the lowest hardware resource compared to other designs with higher precision. The normalized hardware cost for 16-bit and 32-bit designs are similar, and the 128-bit design spends around 26× the FPGA resources when compared to the 32-bit implementation.

### 6.5. Comparison of Related Work

As an implementation with a minimum error–slice–energy cost within all the generated designs, the 32-bit design is applied to compare with other prior works in this section. As shown in Table 4, the hardware cost is summarized in terms of LUTs, FFs, and DSPs. In the last column, the accuracy is further compared between different works.

The number of LUTs and DSPs used in our case study is less than a half compared to [19,26] because of the pipelined structure and three hidden layers used to build the network architecture. The number of convolutional layers and hidden layers used for implementing CNNs in [19,26] are much larger compared to our proposed work. This addition of layers in the network increases the accuracy but utilizes more hardware resources. In contrast, the register count of our design is slightly more than [19] because of the use of a register map to store the values of hidden neurons, which increases the parallelism to execute the computation.

In [27], the design uses a block floating point method of implementation, which consumes higher hardware costs. In [28], the authors have designed the CNN structure based on the sigmoid activation function, while, in this paper, the design is based on the ReLU activation function that is computationally less complex and hence uses fewer hardware resources.

While the LUTs in [25,29] are less than half when compared to our proposed work, [29] uses resource multiplexing, wherein the modules are reused for different operations cycles, which tends to higher latency. In [29], as an example, digit recognition requires 68,139 clock cycles. In [25], the implementation does not follow resource multiplexing; however, it requires many more registers to buffer the data between different layers, and more DSPs are needed for the arithmetic operations.

The accuracy shown in the fifth column demonstrates that our proposed 32-bit design achieves a higher accuracy than that of the implementations with lower precision, including the 8-bit fixed number implementation in [27], 16-bit fixed design in [29], and the 18-bit design in [25]. The design with more resource cost in [19] achieves a higher accuracy compared with that of our proposed architecture, which obtains an overall accuracy of 98.39%.

## 7. Conclusions

This paper presents a parameterizable design for CNNs that can accommodate various design structures and precision levels. Our proposed approach is scalable, allowing for the creation of RTL designs that meet different design specifications. The resulting Verilog code is both synthesizable and implemented in an FPGA demonstration. As a case study, a three-hidden-layer design structure is ultimately implemented and evaluated on an FPGA, achieving an overall accuracy of 98.39% for 32-bit FP precision. The experimental results demonstrate that our design has lower hardware costs than many existing approaches while still achieving reliable accuracy.

## Figures and Tables

**Table 1:** Static analysis of different design structures.

Layer	FP_MUL	FP_ADD	FP_DIV	FP_COMP	Latency
SW = 9, Max	45	45	0	57	100
SW = 3, Max	39	41	0	57	200
SW = 9, Mean	45	93	16	9	103
SW = 3, Mean	39	89	16	9	203

**Table 2:** Resource utilization of different precision designs.

Precision	CLB LUTs	CLB Registers	CARRY8	DSPs	Accuracy
16-bit	9808	3250	228	57	97.07
32-bit	25,848	2677	816	114	98.39
64-bit	86,802	5436	2943	513	99.25
128-bit	424,723	26,955	42,369	1959	99.76

**Table 3:** Energy consumption of different precision designs.

	16-bit	32-bit	64-bit	128-bit
Energy (mJ)	0.28	1.044	2.462	7.867

**Table 4:** Comparison with prior works.

Comparison	LUTs	FFs	DSPs	Accuracy (%)	Quantization Strategy
[19]	55,466	2493	1645	99.17	32-bit floating
[26]	186,251	205,704	2240	-	32-bit floating
[27]	231,761	14,091	1027	-	8-bit Block floating point
[28]	34K	-	-	89	8-bit fixed
[29]	10,208	8204	17	97.3	16-bit fixed
[25]	12,588	48,765	274	97.57	18-bit fixed
Our case study	25,848	2677	114	98.39	32-bit floating

## Data Availability

The design code is shared on Github.
